# Assessment of Risk of Exposure to *Leishmania* Parasites among Renal Disease Patients from a Renal Unit in a Sri Lankan Endemic Leishmaniasis Focus

**DOI:** 10.3390/pathogens11121553

**Published:** 2022-12-16

**Authors:** Chandrani Menike, Rajeewa Dassanayake, Renu Wickremasinghe, Maheeka Seneviwickrama, Indika De Alwis, Ahmed Abd El Wahed, Shalindra Ranasinghe

**Affiliations:** 1 Department of Parasitology, Faculty of Medical Sciences, University of Sri Jayewardenepura, Gangodawila, Nugegoda 10280, Sri Lanka; 2 Nephrology Unit, Teaching Hospital, Anuradhapura 50000, Sri Lanka; 3 Department of Community Medicine, Faculty of Medical Sciences, University of Sri Jayewardenepura, Gangodawila, Nugegoda 10280, Sri Lanka; 4 Provincial General Hospital, Blood Bank, Ratnapuara 70000, Sri Lanka; 5 Institute for Animal Hygiene and Veterinary Public Health, Leipzig University, 04103 Leipzig, Germany

**Keywords:** anti-*Leishmania* antibodies, renal unit, Sri Lanka

## Abstract

*Leishmania donovani* causes both cutaneous and visceral leishmaniasis (CL and VL) in Sri Lanka, where chronic kidney disease (CKD) and kidney transplant recipients’ (KTR) geographical areas overlap. This study aimed to determine the risk of exposure to *Leishmania* infection among renal patients. This cross-sectional study in a renal unit assessed clinical symptoms and signs of CL and VL in recipients of blood/kidney or immunosuppressives. Sera were tested with *Leishmania*-specific DAT and rK-39 ELISA. There were 170 participants. A total of 84.1% (n = 143) were males (CKD: 101, KTR; 42, mean age 45) and 27 were females (females: CKD: 23, KTR: 4, mean age 39 years). Recipients of blood transfusion/s within last 2 years: 75.9% (CKD: 115, KTR: 14), on immunosuppressive therapy: 34.1% (CKD: 13, KTR: 45). Two CKD patients repeatedly showed clear positive titres (1: 12,800 and 1: 3200) with *Leishmania*-DAT and another two (CKD) became marginally positive with rK39-ELISA. Prevalence of anti-*Leishmania* antibodies: 2.4% (4/170). All four patients were clinically asymptomatic and were recipients of recent blood transfusions. Attributable risk of exposure to *Leishmania* infection through blood transfusions was 0.032, OR 2.99 (95% CI = 0.16 to 56.45, *p* = 0.47). Therefore, routine screening of kidney/blood donors and CKD and KTR patients in Sri Lanka may not be necessary.

## 1. Background

Non-communicable diseases (NCDs), including cardiovascular, respiratory diseases, diabetes, cancer, and chronic kidney disease (CKD), were the cause of 75% of all annual deaths in Sri Lanka in 2020 [1]. CKD-contributing deaths were reported as 11% of the total, although most cardiovascular, diabetes and cancer patients show compromised kidneys and go on to develop CKD of known aetiology [1]. CKD of unknown aetiology (CKDu) is on the rise in Sri Lanka [1]. A cross-sectional study on 30,566 CKD/CKDu diagnosed patients from 2003 to 2017 in the North Central Province in Sri Lanka showed steady increases in incidence between 2003 and 2009 and again between 2016 and 2019, mostly in predominantly male farmers [2]. Although CKDu has been associated with environmental fluoride and metal polluted water, other as yet unidentified factors have been suggested [2]. Infectious diseases, including *Leishmania* infection, would add a burden to NCDs patients, including CKD patients in geographical overlapping regions for leishmaniasis and CKD [1].

Leishmaniasis is a complex of infectious diseases transmitted by the bite of an infected female sandfly. Cutaneous leishmaniasis (CL) due to a naturally attenuated *Leishmania donovani* zymodeme MON-37 (CL-SL) and endogenous visceral leishmaniasis (VL) due to a genetically different *L. donovani* zymodeme MON-37 strain have both been established in Sri Lanka [3,4,5,6,7]. The dry North Central Province and the Southern Districts of Matara and Hambantota, with more than 1000 annual CL cases each, contributed to more than 85% of the total annual CL cases between 2009 and 2019 [8]. CL case numbers have increased in the North Central Province as well as spreading to many other parts of the country, becoming a public health problem [9]. Symptomatic visceral leishmaniasis is usually fatal if untreated [10]. Emerging VL cases (between 2001 and 2013) were also reported from the North Central Province or in patients who had previously visited the area [6,7,11]. 

Visceral leishmaniasis diagnosis is based upon the detection of *Leishmania* amastigote parasites in bone marrow or spleen biopsies. However, invasive biopsy procedures pose significant morbidity and mortality risks and are not recommended for screening [12]. A number of non-invasive rapid sero-diagnostic tests, including a dipstick-based method to detect anti-*Leishmania* antibodies in at-risk groups, have been developed and successfully applied as a more suitable strategy for routine screening in Sri Lankan CL endemic regions [13,14,15,16,17]. 

Transmission of *Leishmania* parasites via blood transfusion or solid organ transplantation (SOT) has also been reported [18,19,20]. In 2014, globally, nearly 100 cases of VL were reported in kidney transplantation recipients (KTR) and the numbers have risen to 119 cases by 2019 [19,20]. Asymptomatic VL (42) and CL (5) in KTR patients were also reported [20]. A recent study showed anti-*Leishmania* antibodies in 32% of 50 randomly selected candidates for kidney transplant (KT) who had a number of dialysis cycles [16]. Similarly, concerns have been raised about the observation that a high percentage of blood donors tested positive with anti-*Leishmania* antibodies in leishmaniasis endemic areas globally [17]. It was also shown that immunosuppression-related factors including post-SOT management, significantly contribute to VL case load [21]. Visceral leishmaniasis in post-transplant and immunosuppressed patients might be/is preventable with appropriate screening. Considering this important aspect, a recent literature review in Brazil with high leishmaniasis patient loads showed that transfusion-screening in blood banks for VL may be necessary; however, they recommend further studies [22]. Further, this study points out that different sensitivity and specificity levels of different screening tools in the market are a limitation to arriving at a definitive diagnosis, and demonstration of the parasite in the bone marrow or spleen still remains the gold standard for diagnosing VL, which are more invasive and risky procedures, again raising the concerns of limitation [22]. Interestingly, there are several case reports describing VL and American cutaneous leishmaniasis contributing to CKD with histopathologically different types of nephropathies [23,24,25]. This makes treatment of VL quite challenging [23].

CKDu has been reported in CL endemic districts [26,27]. In CKDu, CL and VL overlapping regions, a KTR might acquire VL via repeated blood transfusions; via a sandfly bite while in the community with either of the two *L. donovani* MON-37 zymodeme strains circulating in Sri Lanka, which may visceralize or reactivate during immunosuppression; or via the transplanted kidney from an unscreened donor. Similar *Leishmania* infection risk factors might apply to CKD patients. However, most leishmaniasis endemic countries, including Sri Lanka, do not screen their CKD patients nor KT donors or recipients for *Leishmania* infection. Correspondingly, although donor blood is routinely screened for malaria parasites in Sri Lanka, as recommended by the WHO, there have not been WHO recommendations to date for screening high-risk CKD renal patients for VL or CL [10,17]. This apparent inconsistency might be due to no laboratory-based diagnostic screening data.

The aim of this study was to determine the risk of exposure to *Leishmania* parasites using two well-established serological methods in a renal unit in a leishmaniasis endemic area in Sri Lanka, to provide solid prevalence data on whether or not screening would help to reduce morbidity and mortality due to *Leishmania* infection in CKD patients needing blood transfusions and in KTR patients.

## 2. Materials and Methods

### 2.1. Study Population 

This study was carried out at the Renal Unit, Teaching Hospital Anuradhapura (THA), situated in the North Central Province CL endemic area in Sri Lanka. 

### 2.2. Study Design 

This was a cross-sectional study conducted from June to October 2015. A total of 176 patients were screened, of which 170 were enrolled in the study meeting inclusion criteria; patients over the age of 18 years, diagnosed by the consultant nephrologist as having CKD or KT. Having CKD was diagnosed according to the criteria described in the Clinical Practice Guideline for the Evaluation and Management of Chronic Kidney Disease; Kidney Disease Improving Global Outcomes 2013 (KDIGO) [28]. Study patients were either warded or attended clinics at the Renal Unit, THA during the study period, and resided in the North Central Province for more than 6 months [27]. All enrolled patients gave written informed consent. Data and samples were collected in the Renal Unit once every two weeks in a consecutive sampling method until the required sample number was reached. Patients who did not meet the inclusion criteria were excluded. 

### 2.3. Clinical History and Examination 

A pre-tested, interviewer-administered questionnaire was used to collect data. A Medical Officer in the Renal Unit, THA collected relevant history and examined study patients to check for VL (fever for > 2 weeks, loss of weight, loss of appetite, anaemia, hepatosplenomegaly, lymphadenopathy) [12]. Routine investigation reports and diagnostic cards of patients were also used to obtain data. Gender, age, date of the most recent blood transfusion/s (blood transfusion within the last 2 years were considered as a risk factor for transfusion transmission of VL) [17], immunosuppressive treatment for ≥ 2 months duration at the time of the study, and the date of KT were also recorded. The same Medical Officer and his team collected data and samples throughout the study to prevent any administrator-based variation. 

### 2.4. Sample Collection and Processing 

Six ml of venous blood was collected from each patient, serum was separated from 4 mL of blood, transported on ice, and stored at −70 °C in the department of Parasitology, Faculty of Medical Sciences, University of Sri Jayewardenepura for serological investigations. The remaining 2 mL of venous blood was used to prepare thick and thin blood films to exclude malaria parasites (this was to confirm that there are no cross-reactions of malaria occurring with VL-DAT and VL-ELISA) [29,30,31]. All those Giemsa-stained smears were also examined under oil emersion for *Leishmania* amastigotes. 

### 2.5. Sero-Diagnosis

Two serological screening tests: the rK39-ELISA (InBios International Inc, USA) and a direct agglutination test (DAT) (Royal Tropical Institute, KIT Biomedicals, Netherlands) were used with the stored serum samples according to the manufacturer’s guidelines. Two tests were selected for the study to increase the sensitivity of detecting anti-*Leishmania* antibodies since it has been previously reported that the agreement between tests rK39-ELISA and DAT tends to vary when anti-*Leishmania* antibody levels are low [22,32]. 

### 2.6. rK39-ELISA

Kalazar Detect™ ELISA, a “two-step” indirect immunosorbent assay, was used with standard manufacturer-provided controls and unknown test serum samples (1:50 dilution, 100µL). Optical density (OD) values were read at 450 nm (MR 96A ELISA microplate reader, Shenzhen Mindray Bio-Medical Electronics Co., Ltd., Shenzhen, China). A result was read as negative when the OD value was < 0.162, an equivocal value when the OD value was between 0.162 and 0.399, and a positive result at > 0.399 OD. At each read OD value, the ratio between positive and negative controls was obtained for checking the validity of the assay [30]. 

### 2.7. Direct Agglutination Test (DAT) 

Two-fold serial dilutions of sera in dilution buffer {0.9% (w/v) physiological saline and 0.78% (v/v) ß-mercapto-ethanol} were made, starting from 1:100 up to 1: 102,400. The same dilutions were prepared from positive and negative controls supplied by the manufacturer. Assay samples were incubated with reconstituted *Leishmania donovani* 1S promastigotes supplied by the manufacturer for 18–20 hrs at ambient temperatures in a “v” bottom micro-titer plate as instructed by the manufacturer. Plates were visually read at 18–20 hrs of incubation against a white background for the presence of agglutination. Samples yielding positive results were assayed in duplicate the following day for confirmation [31]. 

### 2.8. Sample Size Calculation 

Sample size calculation was determined using Epi Info 7. The assumed prevalence was 0.03, confidence interval 95% and the confidence interval precision was 0.02. The calculated sample number was 168. In this study, we collected samples from 170 enrolled CKD and KT patients from the Renal Unit. 

### 2.9. Analysis of Data 

Frequencies of the demographic layout of the cohort having underlying potential risk factors for acquiring VL (KTR, blood transfusions, on immune suppression) and proportions of anti-*Leishmania* antibody positivity, the attributable risk of exposure, and odds ratio (OR) were calculated. 

## 3. Results 

### 3.1. Demography and Underlying Risk Factors 

Out of 170 patients recruited into the study from the Renal Unit, THA, 84% (n = 143) were males (mean age 45 years, range 19–72 years, CKD: 101, KTR: 42,) and 27 females (mean age 39 years, range 18–62 years, CKD: 23, KTR: 4). The mean age of the total sample was 44.48 (± SD 11.36) years. There were 129 (76%) (CKD: 115, KTR: 14) patients who had received blood transfusions within the last two years, with a mean of 4.04 (SD ± 4.13) months since the last transfusion day (range: 2 weeks to 24 months). 26% (n = 45) of patients had had KT (n = 27 KT within the last two years and n = 18 over 2 years) and 58 (34%) (CKD: 13, KTR: 45) patients were on immunosuppressive therapy for more than 2 months at the time of the study (mean duration on immunosuppressive therapy was 34.96 months (range: 1–132 months).

### 3.2. Clinical Profile Related to Visceral Leishmaniasis 

In this cohort, we did not find any patient/s with a past history of either CL or VL. There were patients with a number of clinical features that could be attributed to VL but none of the patients had “case-defining” clinical features suggestive of VL as described by WHO [12] (Table 1). There were 40 patients (23.53%) with anaemia (Hb < 13 g/dl in males and <12 g/dl in females) [33] and this was attributed to renal pathology since there were no other typical clinical features suggestive of VL. Similarly, hepatomegaly (2%) and hepatosplenomegaly (0.6%) were also attributed to consequences of CKD by the nephrologist. These patients were negative with both VL serological tests in our study. Considering the risk–benefit ratio for these liver-affected patients, neither bone marrow nor splenic biopsies were extracted. There was one patient with a skin lesion on the hand who tested negative for CL by the slit-skin smear test. However, biopsy and PCR were not performed since consent was not granted. 

### 3.3. Giemsa-Stained Blood Examination 

Giemsa-stained thick and thin blood smears were negative for malaria in all 170 patients and none had any *Leishmania* amastigotes. 

### 3.4. rK-39 ELISA and DAT VL Diagnostic Tests 

**Figure 1:**
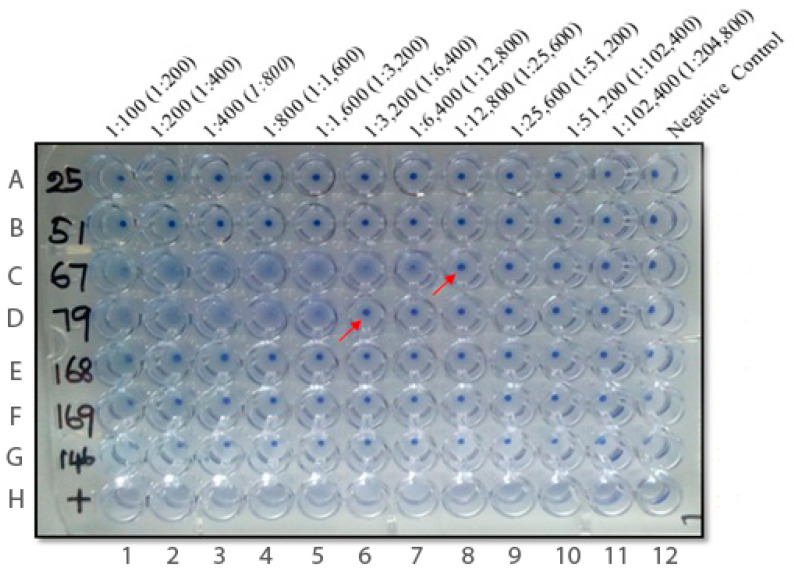
**Repeat DAT results: DAT positive, ELISA positive and some ELISA equivocal samples.** There were two positive samples with end titres 1:12,800 (sample no. 67) and 1:3200 (sample no. 79) that are indicated with arrows. Last row (H1–12) contain positive controls (no agglutination). A-12 to G-12 negative control (agglutination present; blue dot). None of the ELISA positive samples (51 and 169) or ELISA equivocal samples (i.e; 25, 168, 146; only 3 equivocal ELISA samples are shown here) were positive by DAT. Dilutions shown without brackets include the dilutions of patients’ serum samples and ones within brackets indicate the dilution of patients’ serum and antigen together [31].

**Figure 2:**
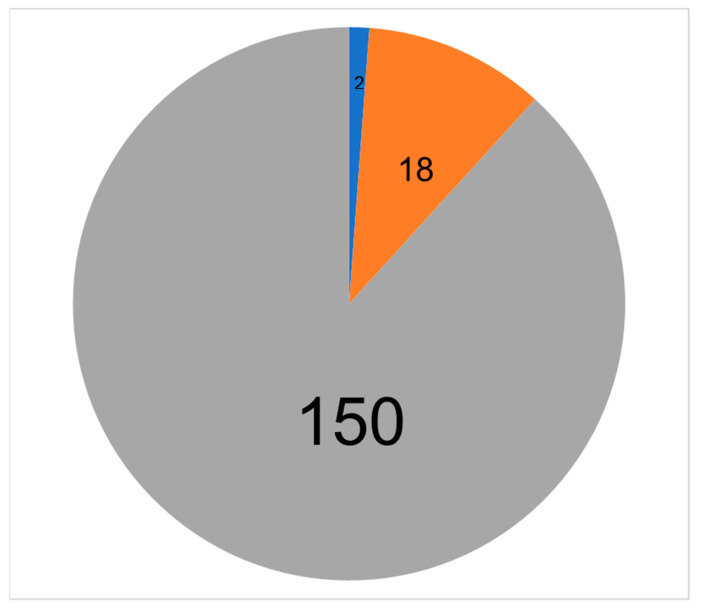
**Collective results of the rK-39 ELISA.** Only 2 samples were clear positive (blue), most of the samples are either negative (gray) or equivocal (orange).

In the laboratory investigations conducted to assess the prevalence of anti-*Leishmania* antibodies, there were two patients’ samples that repeatedly became clearly positive with DAT at dilutions of serum 1:12,800 and 1:3200 (testing on two consecutive days gave the same results) (Figure 1). These were considered true positives since the reported sensitivity and specificity of this test are 94% and 100%, respectively [31,34]. There were also two different patients’ sera that became positive with rK39-ELISA (mean OD samples: 0.449 and 0.455) although OD values were lower than for the positive controls (mean OD of positive controls: 0.531, 0.523, respectively) (Figure 2). These two ELISA-positive patients were also considered as true positives with low antibody titres because the tested values were above the recommended positive OD value of 0.399, which was the cutoff value for a positive test result as recommended by the manufacturer. However, the four study patients with either DAT or ELISA VL positive results did not display clinical features suggestive of active VL [12]. Further invasive bone marrow biopsy procedures were not conducted at the time of the study [12]. There were also a total of 18 patients’ sera that became equivocal with ELISA. However, none of them became positive with DAT or had clinical features suggestive of VL. 

### 3.5. Data Analysis (Prevalence Calculation)

Considering the four patients {DAT (n = 2) and ELISA (n = 2)} as true asymptomatic positives, the calculated proportion of anti-*Leishmania* antibodies in the screened community was 2.4% (4/170). Considering the exposure to risk factors, all four of these patients had received blood transfusions, but were not on any immunosuppressive therapy or were not in the KTR group (only CKD patients). The calculated attributable risk of exposure to *Leishmania* infection through blood transfusions was 0.032, with an OR of 2.99 (95% CI = 0.16 to 56.45, *p* = 0.47) which was not statistically significant.

## 4. Discussion

Recently, the correlation between SOT, immunosuppression, blood transfusions and VL has been widely reported, raising the importance of screening at-risk CKD/and patients awaiting KT [16,17,18,19,20]. However, blood transfusion was not found to be a major risk of acquiring VL in a recent study conducted in the Spanish Balearic Islands (where high rates of asymptomatic VL blood donors have also been reported), perhaps due to low parasite loads [35]. In contrast, a more recent study in Brazil detected anti-*Leishmania* antibodies in 32% (16/50) of candidates awaiting KT who had received multiple transfusions and dialysis sessions, raising concerns of VL as an emerging challenge for KT [17,21]. Our study also showed anti-*Leishmania* antibodies in the blood transfusion group of asymptomatic patients with CKD and supports the notion of *Leishmania* infection as an emerging challenge in renal patients globally. However, when focusing on the local situation, on the contrary, we could argue that the low number of asymptomatic VL among CKD recipients of blood is not a major risk factor for acquiring transfusion transmitted VL. The four *Leishmania* sero-positive patients in our renal cohort had the last blood transfusion 1 and 9 months before (DAT), and 3 and 5 months (ELISA) before, being enrolled in the study. None of these four *Leishmania*-infected renal patients was on immunosuppressive therapy. Therefore, immunosuppression was not a confounding factor for becoming symptomatic in these four patients.

However, it is important to consider immunosuppressive therapy for evaluating the prevalence of specific antibodies, since immunosuppressive therapy is known to interfere with anti-*Leishmania* antibody production and it might give rise to opportunistic VL transmission or to re-activation of asymptomatic VL [21]. 

We report here two patients positive by DAT and two different patients positive by rK-39 ELISA from the high-risk renal patient study group tested for VL. Patients testing positive by only one of these two well-established VL diagnostic serological tests have been previously reported and proposed to be due to different antigen domains used by each of the two tests [22,35,36,37]. We used both tests for each patient, aiming to increase the sensitivity of *Leishmania* detection. In this renal cohort, a 2.4% prevalence of asymptomatic *Leishmania* infection was found. This may be due to the relatively low VL prevalence reported for the North Central Province in Sri Lanka so far [8,13]. Using two well-established diagnostic tests with high sensitivity and specificity; DAT (94% and 100%) and ELISA (99% and 100%), respectively, improved the quality of the data in our study [30,31,34,35,38,39]. 

Whether the 2.4% sero-prevalence of VL is due to blood transfusion or due to exposure to sandfly bites was not further investigated in our study. Assuming that all patients were equally exposed to sandflies, as they were from the same geographic area, if the VL exposure was due to blood transfusion, the attributable risk of *Leishmania* infection through blood transfusion was estimated to be 0.032 with an odds ratio of 2.99 (95% CI = 0.16 to 56.45, *p* = 0.47), which is statistically non-significant. Since the prevalence of asymptomatic VL is not a statistically significant public health problem in Sri Lanka, it may not be necessary to routinely screen blood donors for VL. Although one patient’s test cost would be approximately $ 28 when considering island-wide routine screening of blood donors, this may not be cost effective for the Sri Lankan Ministry of Health to implement at this stage.

However, considering the risk of acquiring transfusion transmission VL and the loss that could occur to an individual patient, it would be useful to perform random testing of blood donors from VL endemic areas in the country to assess the asymptomatic infection rates in the community so that adequate preventive steps could be taken based on the need. 

## 5. Conclusions

Since we detected a 2.4% seroprevalence of anti-*Leishmania* antibodies among 170 CKD/KT patients in one of the CKD-CL/VL overlapping geographic areas in Sri Lanka, more assessment studies of this kind are needed, employing more samples from different endemic areas of the country, to draw nationally important conclusions on whether or not to perform routine screening of renal and blood donors in CL/VL endemic areas in Sri Lanka. 

## Figures and Tables

**Table 1:** Categorization of patients with clinical features.

Clinical Feature	Male (n) (%)	Female (n) (%)
* C/o of un explained fever	4	-
Pallor	27	4
Lymphadenopathy	4	2
C/o Fever + Pallor Hepatomegaly Hepatosplenomegaly	3 4 1	1 - -

* C/o: complained of unexplained fever by patient, but was afebrile on examination and in fever charts. -: none.

## Data Availability

Not applicable.
